# Comparison of sodA and 16S rDNA sequencing for accurate species-level identification of viridans group streptococci (VGS) isolated from patients with infective endocarditis

**DOI:** 10.1186/1471-2334-12-S1-P73

**Published:** 2012-05-04

**Authors:** V Naveenkumar, A Nisha, Thangam Menon

**Affiliations:** 1 Dept. of Microbiology, Dr ALM PG Institute of Basic Medical Sciences, University of Madras, Chennai, India

## Background

Viridans group streptococci (VGS) are commensal flora of the upper respiratory tract in humans and can cause serious infections, like infective endocarditis, septicemia, and meningitis. Accurate identification of VGS to the species level is difficult because they share many physiological characteristics. We report a study using sequencing of 16S ribosomal DNA (rDNA) gene and *sodA* gene to discriminate VGS up to species level.

## Materials and methods

Forty-eight strains of VGS isolated from blood cultures of patients with IE were speciated using *16S rDNA* and *sodA* gene sequencing.

## Results

The isolates were biochemically identified as mitis group (36), salivarius group (9), mutans group (1), and anginosus group (1) and one unidentified species. Based on 16S rDNA sequencing, the strains were identified as S*. sanguinus* (10), *S. oralis* (9), *S.mitis* (7), *S. gordonii* (6), *S. mitis/oralis* (*4*), *S. parasanguinis* (3), *S. sanguinus/oralis* (3), *S. sanguinus/mitis* (1), and one each of *S. mutans* and *S.anginosus.* 27 strains were identified as *S.oralis* by sodA sequencing which included all the seven *S.mitis* strains, seven strains which gave ambiguous results and three strains which were not identified by *16S rDNA* sequencing. Other species such as S*. sanguinus*, *S. gordoni*, *S. parasanguinis*, *S. mutans* and *S. anginosus* were identified by both *16S rDNA* and *sodA* genes.

## Conclusion

Identification of VGS upto species level is difficult using phenotypic characteristics alone. *16S rDNA* sequence analysis was found to be less reliable than *sodA* sequencing for the identification of closely related species such as *S. mitis* and *S. oralis*.

